# Editorial: Clinical implementation of the DSM-5 cultural formulation interview

**DOI:** 10.3389/fpsyt.2024.1520122

**Published:** 2024-12-13

**Authors:** Sofie Bäärnhielm, Hans Rohlof, Valerie DeMarinis

**Affiliations:** ^1^ Centre for Psychiatry Research, Department of Clinical Neuroscience, Karolinska Institutet, Stockholm Health Care Services, Region Stockholm, Stockholm, Sweden; ^2^ Transcultural Center, Region Stockholm, Stockholm, Sweden; ^3^ Outpatient Department for Refugees of Arq National Center for Psychotrauma Treatment, Oegstgeest, Netherlands; ^4^ Research Center for Existential Health, Innlandet Hospital Trust, Hamar, Norway; ^5^ Department of Public Health and Clinical Medicine, Umeå University, Umeå, Sweden

**Keywords:** cultural formulation interview, psychiatric assessment, DSM-5, person-centered, psychiatric diagnosing

There is an increasing need for mental health care to adapt assessment and treatment to cultural and social variety of populations. If cultural factors are not properly assessed, patients may receive an incorrect diagnosis, or the severity of their condition may be misjudged (1). Further, a lack of clinicians’ cultural sensitivity can create communication barriers leading to a patient’s breakdown of trust in the therapeutic process and a hesitancy to communicate important information (2). The introduction of the Cultural Formulation Interview (CFI) in DSM-5 in 2013 (3) involved a breakthrough for improving cultural sensitivity in mental health care with a clinical model and method to enhance cultural awareness in psychiatric assessment based on a person-centered, non-stereotyping approach. Nussbaum (4) defined the CFI as the most person- centered portion of DSM-5 and pointed to the necessity to understand the patient as a person before defining a mental disorder. Already in DSM-IV the core elements of the CFI were introduced as an Outline for Cultural Formulation Interview (OCF), however without instructions on clinical application (5). Aggarwal et al. point at the fact that the CFI emerged from a confluence of approaches from social medicine and interpretive anthropology. To this can be added that the CFI has also roots in the psychodynamic idiographic formulation of exploring different layers of interaction between patient and cultural environment (6). The OCF has been described as a guide for a “mini-ethnographic” narrative assessment (7). Ethnography emphasizes the importance of trying to understand the other’s point of view (8) with the term “thick description” (8) emphasizing the way that ethnography can provide rich and detailed accounts of the social world under study.

The CFI has been implemented widely internationally (9, 10). A recent scoping review on CFI research (11) concludes that the CFI is a useful tool in a variety of settings throughout the world. The CFI was found to increase rapport between patients and clinicians, aid in diagnostic and treatment planning, and increase the subjective exploration of the patient’s illness narrative. Furthermore, the CFI is suggested to have a positive impact on medical communication. However, the authors (11) also identify important gaps in the literature. They note that a large proportion (43%) of existing research comes from the international DSM-5 field trials involving the same database of participants and research teams and underscores the need for research in a variety of clinical contexts and with a variety in clinical populations to solidify the CFI’s use as a valid cross-cultural assessment tool. With this Research Topic on CFI in Frontiers of Psychiatry, we aim to help fill the current research gap on CFI by presenting a variety of CFI research from different contexts, research teams, and populations.

The research presented in this initial Research Topic on the CFI provides different perspectives on the clinical implementation of the CFI in diverse settings, populations, and geographical contexts. The studies give a broad view on applications of the CFI, how the CFI functions among diverse populations as well as discussing adaptations and needs for further refinement. The articles mirror how the CFI has started to be used outside of the initial scope of supporting clinical cultural-sensitive psychiatric assessments, to be an inspiring approach for exploring the social and cultural world of the other, to get a “thick descriptions” in different clinical encounters and through other research study applications.

Hadding et al. explore the experiences of acculturation into secular Swedish society of former cult members. In the study the CFI is used as a semi-structured interview guide. The authors suggest the need for support for former cult members from the healthcare system, especially regarding mental health while establishing themselves into mainstream society. In another Swedish study Ioannou et al. use the CFI for conceptualizing a Cultural Concept of Distress. Through analyzing case studies with patients with bipolar disorders they argue that the concept of “highly sensitive person” can be approached as a Cultural Concept of Distress. A comprehensive project on implementing the CFI in the Netherlands is reported in the study by (Silvius et al.). The authors conclude that implementation of the CFI requires a fundamental rethinking of the intake assessment, shifting it from a symptom-oriented approach towards a context- and person-centered one. They further suggest including training with the CFI to be included within a broader cultural-competency training.

The debate about whether social medicine approaches like (“structural competence”) should be emphasized over interpretive medical anthropology approaches (glossed as “cultural competence”) is deepened in the study of (Aggarwal et al.). The authors analyze how patients responded to CFI questions on social stressors, supports, barriers to help seeking, and access to care, and an emerging framework on the social determinants of health. They found that social determinants of mental health can be elicited through the CFI and discuss whether the cultural formulation approach would benefit from additional revision to facilitate assessment of socio-structural factors. Claus et al. present a study protocol on a planned mixed-methods study evaluating the value of the CFI in the psychiatric assessment of young asylum seekers in Belgium. The potential benefits and challenges of utilizing the CFI in the assessment of eating disorders is explored by (Strand et al.). The authors suggest the CFI be used as a standard tool in specialist eating disorder services. Wallin et al. explore what information the DSM-5 CFI revealed when used with native Swedish-speaking patients as part of routine clinical psychiatric assessment at an outpatient clinic and in an another with non-native speaking patients (Wallin et al.). The authors suggest a further refinement of the CFI, through re-framing of the identity questions so that they are more easily understood.

Hopefully this variety in research will result in a broad clinical implementation of the CFI and in evaluation research.
